# Supplementary material for: Elevated Na is a dynamic and reversible modulator of mitochondrial metabolism in the heart
Source: Nat Commun. 2024 May 20;15:4277. doi: 10.1038/s41467-024-48474-z (PMC11106256; doi:10.1038/s41467-024-48474-z)
Supplement: Supplementary file 1 — Supplementary Information [file 41467_2024_48474_MOESM1_ESM.pdf]

Supplementary Information:

Elevated Na is a Dynamic and Reversible Modulator of Mitochondrial Metabolism in the Heart

Chung YJ, Hoare Z, Baark F, Yu CS, Guo J, Fuller W, Southworth R, Katschinski D, Murphy MP, Eykyn TR, Shattock MJ.

Supplementary Discussion: Total adenine pool during Na elevation:

The total adenine nucleotide pool (TAN) (ATP+ADP+AMP) was estimated in extracted tissue snap frozen at the end of the respective ^{31}P experiments using ^{1}H NMR (Figure S1). TAN was not significantly different at 40 mins (Na elevation) compared to its time-matched control. The TAN pool was also not significantly different at 60 mins (ouabain washout) with respect to its time-matched control.

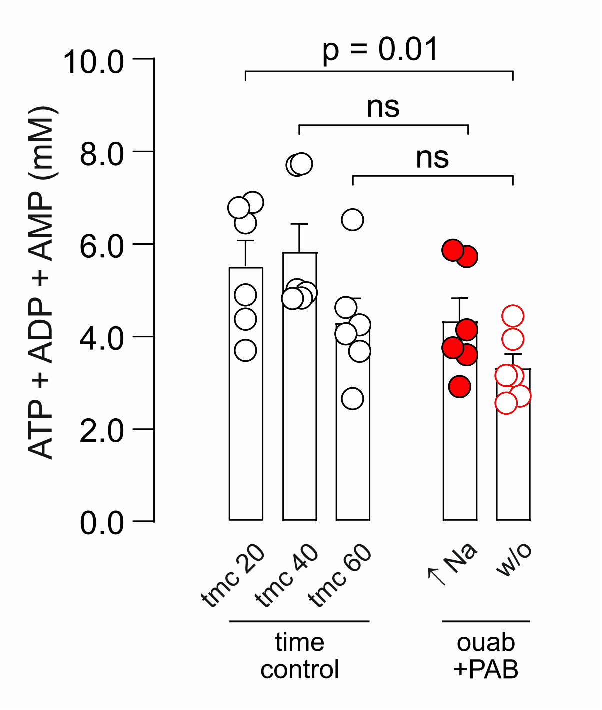

Figure S1: Total adenine nucleotide pool (TAN) (ATP+ADP+AMP) estimated in extracted tissue snap frozen at the end of the respective ^{31}P experiments using ^{1}H NMR.

However, we note a trend towards lower TAN during elevated Na compared to baseline which was further depressed at washout. While the time-matched control is unchanged at 40 mins, and maybe marginally lower at 60 mins, this is not statistically significant. Given the variability of these data we are not able to rule out the possibility that the TAN pool is decreased by elevated Na, and this could offer a plausible explanation why we observed decreased ATP with unchanged PCr.

Magnetisation Transfer Experiments:

Under conditions of Na elevation, we see a decrease in magnetization transfer from Pi to ATP. However, as Pi is higher, then the *total flux*, given by the product of the rate constant x concentration (peak area), could be conserved. To address this, we have analysed the *total flux* by multiplying the magnetization transfer ΔM_z by Pi (expressed relative to baseline) (see Figure S2).

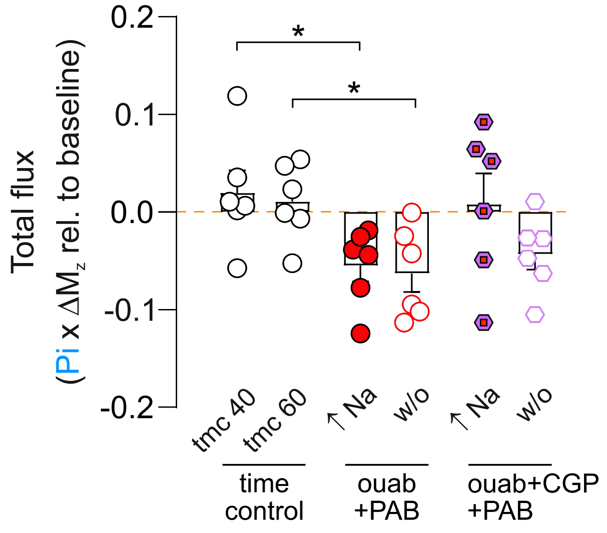

Figure S2: Total magnetization transfer flux from Pi to ATP measured as the product of the rate constant x Pi concentration (peak area).

Under conditions of high Na, *total flux* remains decreased and, interestingly, this reduction is sustained during washout. This recapitulates the findings for ATP during high Na and washout shown in Figure 2a. This is not observed for the time-matched control nor for the CGP treated hearts. Thus, supporting the proposal that ATP synthesis is reduced during Na elevation.
