# Supplementary material for: Elevated Na is a dynamic and reversible modulator of mitochondrial metabolism in the heart
Source: Nat Commun. 2024 May 20;15:4277. doi: 10.1038/s41467-024-48474-z (PMC11106256; doi:10.1038/s41467-024-48474-z)
Supplement: Supplementary file 3 — Reporting Summary [file 41467_2024_48474_MOESM3_ESM.pdf]

nature portfolio

Corresponding author(s): Shattock MJ, Eykyn TR

Last updated by author(s): 15/04/2024

Reporting Summary

Nature Portfolio wishes to improve the reproducibility of the work that we publish. This form provides structure for consistency and transparency in reporting. For further information on Nature Portfolio policies, see our Editorial Policies and the Editorial Policy Checklist.

Statistics

For all statistical analyses, confirm that the following items are present in the figure legend, table legend, main text, or Methods section.

n/a	Confirmed	
☐	☒	The exact sample size (*n*) for each experimental group/condition, given as a discrete number and unit of measurement
☐	☒	A statement on whether measurements were taken from distinct samples or whether the same sample was measured repeatedly
☐	☒	The statistical test(s) used AND whether they are one- or two-sided<br>*Only common tests should be described solely by name; describe more complex techniques in the Methods section.*
☐	☒	A description of all covariates tested
☐	☒	A description of any assumptions or corrections, such as tests of normality and adjustment for multiple comparisons
☐	☒	A full description of the statistical parameters including central tendency (e.g. means) or other basic estimates (e.g. regression coefficient) AND variation (e.g. standard deviation) or associated estimates of uncertainty (e.g. confidence intervals)
☐	☒	For null hypothesis testing, the test statistic (e.g. *F*, *t*, *r*) with confidence intervals, effect sizes, degrees of freedom and *P* value noted<br>*Give P values as exact values whenever suitable.*
☒	☐	For Bayesian analysis, information on the choice of priors and Markov chain Monte Carlo settings
☒	☐	For hierarchical and complex designs, identification of the appropriate level for tests and full reporting of outcomes
☒	☐	Estimates of effect sizes (e.g. Cohen's *d*, Pearson's *r*), indicating how they were calculated

Our web collection on statistics for biologists contains articles on many of the points above.

Software and code

Policy information about availability of computer code

Data collection	No customised software or algorithms were used in the collection of the data
Data analysis	No customised software or algorithms were used in the analysis of the data

For manuscripts utilizing custom algorithms or software that are central to the research but not yet described in published literature, software must be made available to editors and reviewers. We strongly encourage code deposition in a community repository (e.g. GitHub). See the Nature Portfolio guidelines for submitting code & software for further information.

Data

Policy information about availability of data

All manuscripts must include a data availability statement. This statement should provide the following information, where applicable:

- Accession codes, unique identifiers, or web links for publicly available datasets
- A description of any restrictions on data availability
- For clinical datasets or third party data, please ensure that the statement adheres to our policy

All data, without restriction, are available on request from the corresponding author. A Source Data file is provided as supplementary material

Research involving human participants, their data, or biological material

Policy information about studies with human participants or human data. See also policy information about sex, gender (identity/presentation), and sexual orientation and race, ethnicity and racism.

Reporting on sex and gender	N/A
Reporting on race, ethnicity, or other socially relevant groupings	N/A
Population characteristics	N/A
Recruitment	N/A
Ethics oversight	N/A

Note that full information on the approval of the study protocol must also be provided in the manuscript.

Field-specific reporting

Please select the one below that is the best fit for your research. If you are not sure, read the appropriate sections before making your selection.

☒ Life sciences ☐ Behavioural & social sciences ☐ Ecological, evolutionary & environmental sciences

For a reference copy of the document with all sections, see nature.com/documents/nr-reporting-summary-flat.pdf

Life sciences study design

All studies must disclose on these points even when the disclosure is negative.

Sample size	Power analysis indicates for our expected data that to observe a 25% difference between groups, the sample size for a variable with a coefficient of variation of ~0.1 requires 6 hearts/observations per group (using a 2-tailed t-test). This will ensure a less than 0.05 probability that the 'difference' is false (type I error) or that a real difference is not detected (type II error). For experiments where 1 sample represents one well of cells in culture, power calculations are based on the type of data that, in our experience, shows the most variability – protein abundance via Western blotting (standard deviation ~20% mean). For comparison of 2 groups we require 6 observations per group to detect a 40% difference between groups with a power of 0.8 (t-test). For comparison of multiple groups we require 7 observations per group in order to detect a 40% difference between means using ANOVA. While it is not possible to perform power analysis on all proteins in a metabolomics study, a general assumption of 50% variation with 2-fold difference with a power of 0.80 and alpha of 0.05 will require a group size of 4. Our own experience with quantitative metabolomic analysis of cardiac samples indicates 4 high quality biological replicates will detect 80% of metabolites changing 2-fold or more.
Data exclusions	None
Replication	All experiments were replicated based on the power analysis above.
Randomization	All experiments were preformed in a pseudo randomised manner where controls and treatments groups were contemporaneous and on any given day both control and treatment groups would be studied.
Blinding	Where possible (ie sample analysis, metabolomics etc) all samples were blinded to the experimenter. In some heart perfusions blinding was not possible as blebbistatin was actively and iteratively titrated against contractility in the active ouabain treatment arms making blinding impossible.

Reporting for specific materials, systems and methods

We require information from authors about some types of materials, experimental systems and methods used in many studies. Here, indicate whether each material, system or method listed is relevant to your study. If you are not sure if a list item applies to your research, read the appropriate section before selecting a response.

Materials & experimental systems

n/a	Involved in the study
☐	☒ Antibodies
☒	☐ Eukaryotic cell lines
☒	☐ Palaeontology and archaeology
☐	☒ Animals and other organisms
☒	☐ Clinical data
☒	☐ Dual use research of concern
☒	☐ Plants

Methods

n/a	Involved in the study
☒	☐ ChIP-seq
☒	☐ Flow cytometry
☒	☐ MRI-based neuroimaging

Antibodies

Antibodies used

Rabbit polyclonal anti-HIF-1 alpha antibody (Novus, NB100-479); Rabbit monoclonal anti-GAPDH antibody (Abcam, EPR16891 Goat anti-rabbit-HRP (Santa Cruz, sc-2030)

Validation

HIF-1 alpha antibody validated by manufacturer in cells exposed to chronic hypoxia (https://www.novusbio.com/products/hif-1-alpha-antibody_nb100-479). anti-GAPDH antibody validated by manufacturer in a wide range of cell lines and animal tissues, including rat (https://www.abcam.com/products/primary-antibodies/gapdh-antibody-epr16891-loading-control-ab181602.html).

Animals and other research organisms

Policy information about studies involving animals; ARRIVE guidelines recommended for reporting animal research, and Sex and Gender in Research

Laboratory animals

Adult male Wistar rats (200-250g: 6-8 weeks of age) and adult male C57b/6N mice (3-4 months of age) were used. All animals were housed according to UK Home Office regulations at 20-24oC, 45-65% humidity and 12h/12h light/dark cycle and this is now specified in the manuscript.

Wild animals

N/A

Reporting on sex

All experiments were only perfomed on male anaimals. Unfortunately our funding provision at the time only supported sufficient animals to study males.

Field-collected samples

Not applicable

Ethics oversight

Animal procedures were performed in compliance with UK Home Office Guidance on the Operation of the Animals (Scientific Procedures) Act of 1986, the Directive 2010/63/EU of the European Parliament, following a registered IACUC protocol (no. T11.2) and the King's College London institutional guidelines.

Note that full information on the approval of the study protocol must also be provided in the manuscript.

Plants

Seed stocks

N/A

Novel plant genotypes

N/A

Authentication

N/A
